# Cellular adhesiveness and cellulolytic capacity in *Anaerolineae* revealed by omics-based genome interpretation

**DOI:** 10.1186/s13068-016-0524-z

**Published:** 2016-05-23

**Authors:** Yu Xia, Yubo Wang, Yi Wang, Francis Y. L. Chin, Tong Zhang

**Affiliations:** Shenzhen Institute of Research and Innovation, The University of Hong Kong, Shenzhen, China; Department of Civil Engineering, The University of Hong Kong, Hong Kong SAR, China; Department of Computer Science, The University of Hong Kong, Hong Kong SAR, China; Department of Computing, Hang Seng Management College, Hong Kong SAR, China; Environmental Biotechnology Lab, The University of Hong Kong, Pokfulam Road, Hong Kong, China

**Keywords:** Cellular adhesion, Aggregation, Cellulolytic capacity, Anaerolineae, Chloroflexi, Anaerolinales

## Abstract

**Background:**

The *Anaerolineae* lineage of *Chloroflexi* had been identified as one of the core microbial populations in anaerobic digesters; however, the ecological role of the *Anaerolineae* remains uncertain due to the scarcity of isolates and annotated genome sequences. Our previous metatranscriptional analysis revealed this prevalent population that showed minimum involvement in the main pathways of cellulose hydrolysis and subsequent methanogenesis in the thermophilic cellulose fermentative consortium (TCF).

**Results:**

In further pursuit, five high-quality curated draft genomes (>98 % completeness) of this population, including two affiliated with the inaccessible lineage of SBR1031, were retrieved by sequence-based multi-dimensional coverage binning. Comparative genomic analyses revealed versatile genetic capabilities for carbohydrate-based fermentative lifestyle including key genes catalyzing cellulose hydrolysis in *Anaerolinea* phylotypes. However, the low transcriptional activities of carbohydrate-active genes (CAGs) excluded cellulolytic capability as the selective advantage for their prevalence in the community. Instead, a substantially active type VI pili (Tfp) assembly was observed. Expression of the tight adherence protein on the Tfp indicated its function for cellular attachment which was further testified to be more likely related to cell aggregation other than cellulose surface adhesion. Meanwhile, this Tfp structure was found not contributing to syntrophic methanogenesis. Members of the SBR1031 encoded key genes for acetogenic dehydrogenation that may allow ethanol to be used as a carbon source.

**Conclusion:**

The common prevalence of *Anaerolineae* in anaerobic digesters should be originated from advantageous cellular adhesiveness enabled by Tfp assembly other than its potential as cellulose degrader or anaerobic syntrophs.

**Electronic supplementary material:**

The online version of this article (doi:10.1186/s13068-016-0524-z) contains supplementary material, which is available to authorized users.

## Background

Anaerobic digestion, as a key environmental technology for resource recovery, is empowered by microbial reactions involving a complex community. *Anaerolineae* (also known as subphylum I of *Chloroflexi* phylum) had been identified as one of the core populations, and for most of the cases, the dominating proportion of anaerobic digestive systems [1, 2]; however, its roles in anaerobic digestion process remain uncertain due to a general lack of sequenced genomes.

A series of metabolic reactions such as hydrolysis, acidogenesis (fermentation), acetogenesis, and methanogenesis are involved in the process of anaerobic digestion. Normally, the *Anaerolineae* linage has been regarded as a typical fermentative population within the community. As hydrogen could be produced during fermentation of soluble sugar, researchers also speculated that *Anaerolineae* acted as anaerobic syntrophs which conduct reverse electron transfer via tightly coupled mutualistic interaction with methanogens [1]; however the validity of *Anaerolineae* in syntrophic methanogenesis is not yet confirmed. Additionally, the common ability to grow on starch (alpha-glucan polysaccharides) [3, 4] and the recent discovery of cellulolytic representative *Ornatilinea apprima* [5] is attracting increasing research interest on the importance of this lineage in the bottlenecking polysaccharide hydrolysis step of anaerobic digestion. Moreover, the flow velocity within the digester, especially in upflow anaerobic sludge blanket reactor (UASB), may select organisms which can adhere to each other to form well-settling granular sludge. This widely distributed *Anaerolineae* population had been reported as both the backbone of sludge granules [6] and the causative agent of filamentous bulking in UASB [2, 7].

Currently ten strains had been isolated in *Anaerolineae* class [3–5, 8–11]. These representative strains isolated from anaerobic sludge treating various pollutants help to resolve the phylogenetic composition of this lineage into eight genera representing one family of *Anaerolineaceae* in one single order of *Anaerolineales*. Phenotypic comparison of the cultivated strains identifies a number of common traits including filamentous morphology as well as non-motile, non-sporulation, and gram-negative characteristics [3–5, 8–11]. In contrast, genomic information is quite limited for this class; indeed there is only one finished complete genome, *Anaerolinea thermophila* UNI-1 (short as UNI-1 in subsequent discussion), available in IMG 4.0 (up to 12 Jan 2016). Further recovery of interpretative genomes is requisite to disclose the ecological importance of *Anaerolineae* as a core population in anaerobic digestion process.

Rapid accumulation of next generation sequencing (NGS) data from various metagenomes had made genome reconstruction independent from isolation possible [12–15]. Such cultivation-independent binning method based on multi-dimensional abundance profile had provided initial genomic insight of the metabolic styles of the previously inaccessible phyla-like TM6 and OP8 etc. [15–17]. This creative approach had helped to expand the evolutionary boundaries of *Dehalococcoidia* lineage of the *Chloroflexi* from obligate organohalide respiration to fermentation, CO_2_ fixation, and acetogenesis [18, 19]; however, these frontier work did not emphasize on the lineage of *Anaerolineae* which is important in the operation of anaerobic reactors.

In the present study, with a purpose to resolve the special physiochemical features that support accumulation of *Anaerolineae* in the thermophilic cellulose-fermenting reactor, we utilized a sequence-based metagenomic binning to recover high-quality genomes from *Anaerolineae* lineage. Comparative genomics were conducted to reveal specific genetic traits related with key functions in anaerobic digestion. Beneficial ecological functions of *Anaerolineae* within the community were inferred based on the expressed genes and pathways identified by metatranscriptomic sequencing and then testified in experiments. Information obtained here would add a great amount of contextual information to the ecological importance of *Anaerolineae* in anaerobic digestive systems and help to resolve the intra-physiological differences among the uncultivated majority of this lineage.

## Results and discussion

### Two-dimensional coverage binning and quality evaluation

Metagenomic DNA extracted from sludge samples collected from the same thermophilic anaerobic cellulose-degrading reactor but at two different time points [the short-term enrichment at 120 days of enrichment (SE) and the long-term enrichment at 549 days (LE)] were deeply sequenced to construct the two differential coverage for genome reconstruction. The 129,535,600 high-quality reads obtained from illumina paired-end sequencing (see Additional file 1: Table S1 for a summary) were de novo assembled, resulting in a total of 119 Mb operational scaffolds (scaffolds longer than 1 kb) with N50 of 19,859 bp. Most of the reads (87 %) were included in the assembly with the longest scaffolds being 640 kb (Additional file 1: Table S2).

**Fig. 1:**
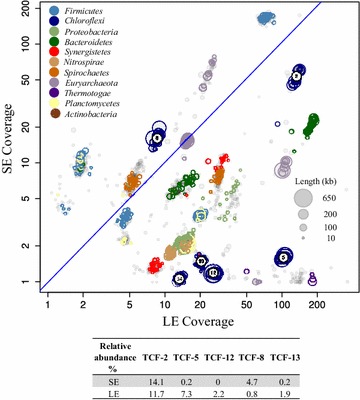
Two-dimensional coverage* plot* of the scaffolds assembled from the short-term enrichment (SE) and long-term enrichment (LE) metagenomes. Size of the* circle* is scaled by the square root of the length of scaffold. And scaffolds are* colored* according to their consensus taxonomic annotation at Phylum level. Only scaffolds longer than 10 kb are depicted.* Diagonal line* in* blue* represents stable coverage between two metagenomes. Scaffolds clustered at close coverage represent potential genome bins and were labeled according to the bin name. The relative abundance of the five retrieved curated genome bins in the SE and LE metagenomes is listed in the table. The relative abundance of each genome bin is estimated as the number of reads mapped to the draft genome in percentage of the total number of reads in the metagenome

As shown in Fig. 1, the coverage binning produced six primary genome bins (named as TCF-2, 5, 8, 12, 13, and 14) showing closely clustered coverage in the SE and LE metagenomes. The accumulation of *Chloroflexi* during enrichment facilitated the retrieving of the related genome bins (Additional file 1: Figure S1). Then, tetra-nucleotide frequency (TNF) was used to filter out possible contamination at hierarchy distance of 0.1 [20]. The quality of these six primary *Chloroflexi* genome bins was then evaluated in terms of genome completeness and contamination, respectively based on the occupation and duplication of the 107 essential single-copy genes (ESCGs) shared >95 % of all bacteria (Additional file 1: Tables S3, S4). Except for TCF-14, the other five genome bins showed comparable completeness and ESCG redundancy to that of the other finished genomes of *Chloroflexi* (completeness larger than 96 % with redundancy less than 5 %, Additional file 1: Table S5). This quality estimation was double checked by an alternative method based on 35 conserved single-copy COGs [21]. Consistent completeness and purity estimation results were observed for these five genome bins (Additional file 1: Table S6). The subsequent genome annotation was only based on these five high-quality genome bins. The other retrieved genomes can be found in Additional file 1: Table S7.

#### Phylogenic position of curated genomes

**Fig. 2:**
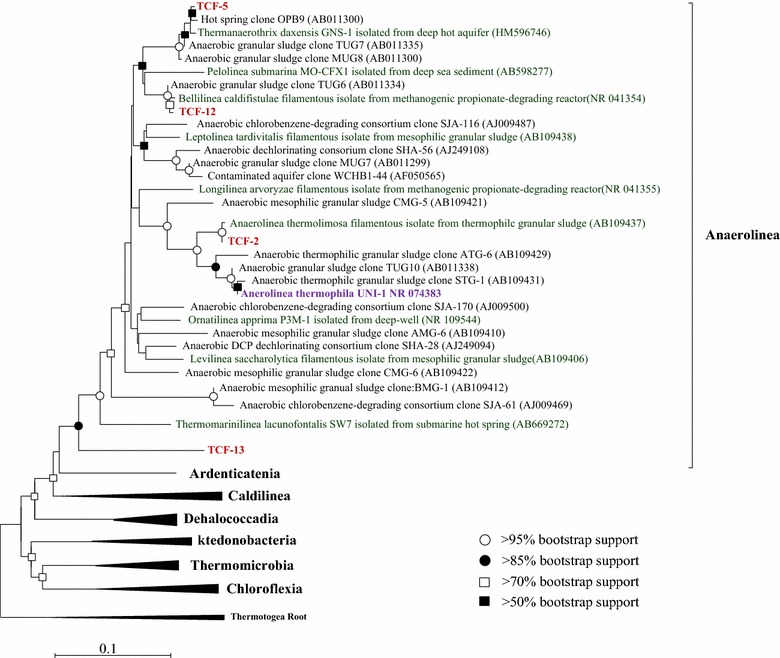
Phylogenetic analysis of the five draft genomes within *Chloroflexi* phylum. Neighbor-joining phylogenetic tree based on *16S rRNA* gene. The tree is produced with neighbor-joining analysis based on ClustalW alignment. *16S rRNA* gene sequences of *Thermotogae* are used to root the tree. Bootstrap value is obtained with the maximum-composite-likelihood methods based on 1000 replicates. Bootstrap values greater than 50 % are indicated at branch points. Branch labels are colored according to their categories (1) our five draft genomes in *red*; (2) complete genomes in *purple*; (3) isolated strains are in *green*

**Fig. 3:**
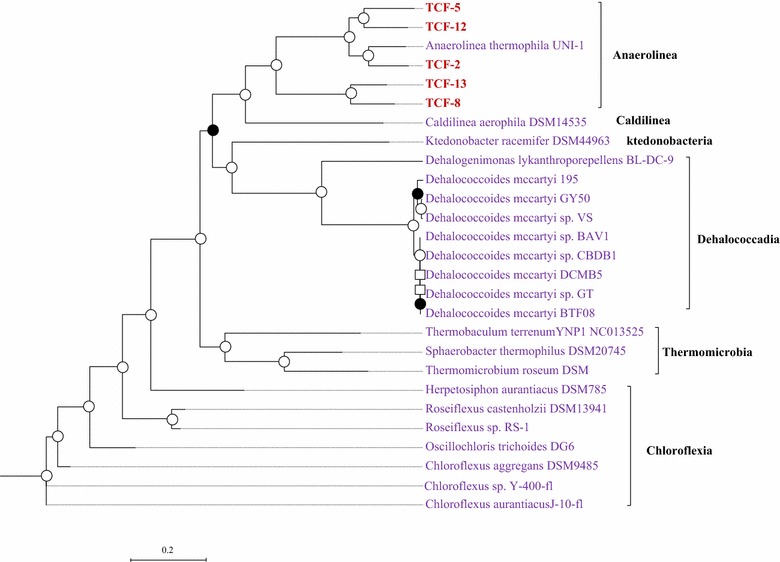
Phylogenetic analysis of the five draft genomes within *Chloroflexi* phylum. Maximum-likelihood tree based on concatenated alignment of 35 essential single-copy genes (ESCGs) conserved in a single-copy manner among five curated genomes retrieved and twenty-two finished genomes within *Chloroflexi* phylum. Default protein model of Phyml 3.1 is used to construct the tree with 100 bootstraps based on MUSCLE alignment. Bootstrap values greater than 50 % are indicated at* branch points*. Branch labels are* colored* according to their categories (1) our five draft genomes in *red*; (2) complete genomes in *purple*; (3) isolated strains are in *green*

To solve the phylogenetic position of these five genome bins, firstly neighbor-joining phylogenetic tree was constructed with recovered *16S rRNA* genes of the TCF-2, 5, 12, and 13 and high-quality 16S clone sequences downloaded from Silva SSU 15.0 database. As shown in Fig. 2, all of the curated genomes were placed within *Anaerolineae* class. TCF-2, TCF-5, and TCF-12 were clustered to the order of *Anaerolinales* and respectively affiliated with *A. thermolimosa*, *Bellilinea caldifistulae*, and *Thermanaerothrix daxensis*. In contrast, TCF-13 showed no confirmed affiliation to any known genus. The closest known relative of TCF-13 is *Thermomarinilinea lacunofontalis* at maximum-likelihood evolutionary distance larger than 0.1, suggesting that this species may belong to a novel lineage within *Anaerolineae* class. This lineage was named as the SHA-31 family of SBR1031 order in the updated greengenes taxonomy (published in May 2013) [22]. Since the *16S rRNA* gene of TCF-8 is too short (only 65 bp) to make confident alignment, maximum-likehood tree based on the concatenated alignment of 35 single-copy ESCGs shared among the five genome bins and twenty-two finished genomes of *Chloroflexi* was used in addition to phylogenetic tree based on *16S rRNA* genes. The concatenated clustering indicated close phylogenetic affiliation of TCF-8 to the SBR1031 lineage containing TCF-13 (Fig. 3). Further comparison based on the average nucleotide identity (ANI) also supported this affiliation that TCF-8 shared far more genes with TCF-13 (811 shared genes) than with any other genomes of the *Anaerolineales* lineage (Additional file 1: Figure S2b). TCF-8 and TCF-13 shall represent different species of this previously inaccessible lineage since ANI between these two genomes (79.3 %) was less than 94 % [23] and in silico DNA–DNA hybridization value (DDH of 17.7 %) much lower than 70 % [24] (Additional file 1: Figure S2a).

#### General physiology and prevalence of Anaerolineae in the TCF community

Among the five curated genomes obtained, TCF-2, 5, and 12 showed average genome size of 3.5 Mb and GC content 54 % which is more consistent with that of *A. thermophila* UNI-1 (the only available complete genome of *Anaerolineae*), while TCF-8 and TCF-13 showed slightly bigger genome (>4.0 Mb) with higher GC content (around 65 %) (Table 1). Resembling their phylogenetic affiliation (Figs. 2, 3), complete-linkage clustering on COG orthologs also indicated that TCF-8 and TCF-13 were functionally divergent from the cluster containing TCF-2, 5, 12, and UNI-1 (Additional file 1: Figure S3). In both the SE and LE metagenome, the order of *Anaerolinales* containing TCF-2, 5, 12, and UNI-1 were generally more prevalent than SBR1031 order containing TCF-8 and TCF-13 (Fig. 1). TCF-2 taking 14.1 % of SE and 11.7 % of LE metagenome was one of the dominant populations within the TCF community. Despite of the large population size, TCF-2 expressed only comparatively small fraction (27.8 % of possible transcripts detected, Table 1) of its genetic complement in situ and this at a moderate level of expression suggesting the tight regulation of gene expression to facilitate preferential metabolism in TCF-2. The metabolic advantage of this population will be discussed from the major steps of anaerobic digestion process: fermentative metabolism, cellulose hydrolysis, and syntrophic methanogenesis.

**Table 1:** Genomic information of the five *Anaerolinea* genomes retrieved from thermophilic cellulose-degrading metagenomes

General information	TCF-2	TCF-5	TCF-12	TCF-8	TCF-13	*A. thermophila* UNI-1
IMG Genome ID	2561511051	2561511052	2561511055	2561511056	2561511053	N/A
Total length (Mb)	3.8	3.0	3.7	4.1	4.0	3.5
Scaffolds (n)	55	27	69	51	153	1
Average sequence length (bp)	71,706	11,837	56,472	84,553	27,370	N/A
GC (%)	54	55	53	64	65	54
Estimated completeness based on ESCG^a^ (%)	99.1	98.1	100.0	99.1	100.0	99.1
Estimated redundancy of ESCGs (%)	3.8	1.9	3.7	2.8	3.7	2.8
Genes	3602	3005	3580	3681	3659	3224
Protein-coding genes	3550	2949	3523	3634	3609	3166
rRNA genes (5S/16S/23S)	2/2/0	1/1/1	1/1/1	3/1/0	1/1/0	2/2/2
tRNA gene (n)	43	45	48	41	43	50
Genes with functional prediction (%)	81.4	83.2	80.4	80.8	80.5	53.7
Genes with transcription (%)	27.8	35.4	18.1	1.8	2.8	N/A
Duplicated AA^b^ (n/ %)	814 (22.9 %)	734 (24.9 %)	921 (26.1 %)	932 (10.8 %)	893 (24.7 %)	757 (23.9 %)

*N/A* data not available

^a^ Completeness estimation based on 107 conserved single-copy genes, named as essential single-copy genes (ESCGs) of >95 % complete bacterial genomes [13]

^b^ Putative duplication among amino acid (AA) sequences within each chromosome (based on BLASTP bitscore ≥70, similarity ≥30 over at least 70 % of the query length [66])

### Fermentative lifestyle of *Anaerolineae*

*Anaerolineae* showed versatile metabolic abilities on carbohydrate fermentation. Glycolysis pathway towards acetate and lactate production was conserved among TCF-2, 5, 12, and UNI-1 (Additional file 1: Figure S4) suggesting that acetate and lactate shall be produced during fermentation. TCF-8 and TCF-13 also processed the complete acetate pathway but not that for lactate generation. Additionally, common encoding-gene cluster of NiFe hydrogenase (COG3260, 3261, 3262) and related proteins in five curated genome bins and UNI-1 indicated the metabolic ability to produce hydrogen during fermentation, consistent with previous experimental results based on isolated strains [3–5, 8–11].

#### Cellulolytic activities

**Fig. 4:**
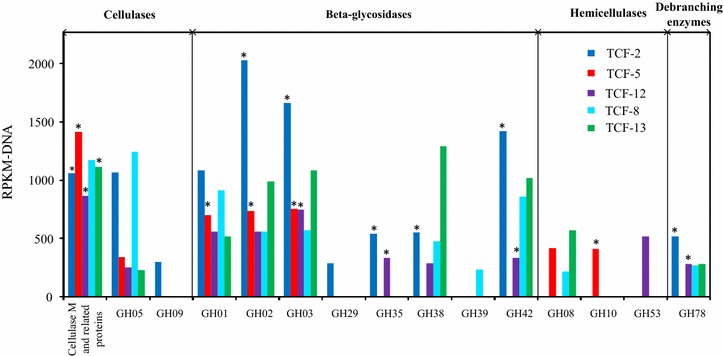
Glycoside hydrolase (GH) families involved in lignocellulose hydrolysis in five curated genomes of *Anaerolineae.* Relative abundance of GH families is measured in terms of RPKM-DNA. GH families showed transcriptional activities are indicated with *asterisk*. GH families are categorized according to the classification proposed by Pope et al. [67]

Except for TCF-8, active transcription of cellulase M and cellulases of GH05 and GH09 in the other four *Anaerolineae* genomes indicated their ecological roles as cellulose hydrolyzers in the cellulolytic community (Fig. 4). We speculated that the *Anaerolineae* populations might rely on extra-cellular cellulase systems for hydrolysis because no cohension, dockerin (key component of cellulosome complex), or any cellulase-related carbohydrate-binding modules (CBMs) could be identified in the five curated genomes and UNI-1. Nevertheless our previous study on the transcriptional characterization of this TCF consortium showed minimum contribution of the *Chloroflexi*, compared to *Clostridiales* and *Bacteroidetes* in overall cellulose hydrolysis [25]. Such transcriptional inefficiency may be regulatory that the cellulase M clusters of *Anaerolineae* were not protected by the preceding heat shock protein like that found in *Clostridiales* [25]. Consequently, cellulolytic capacity is unlikely the driven force for the prevalence of *Anaerolineae* within the community.

#### Major transcription of type IV pili (Tfp)

**Fig. 5:**
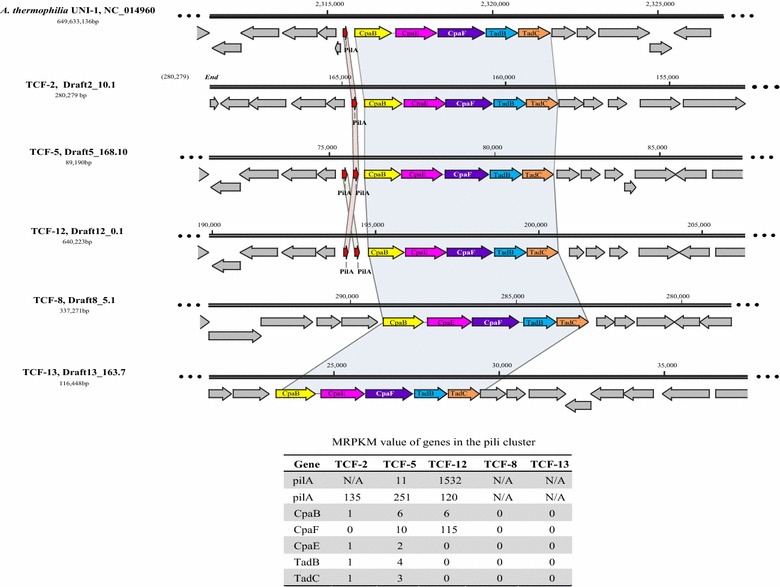
Arrangement of the pili cluster on the genome of the five curated genomes and *A. thermophila* UNI-1. The name and length of scaffold carrying the pili gene cluster is listed to the* left* of the gene arrangement. Genes within the pili cluster are colored according to their homologies that genes show bidirectional best blast match to each other are in the* same color*. *PilA* pilus assembly protein *Flp/PilA*, *CpaB/E/F* pilus assembly protein CpaB/E/F,* TadB/C* tight adherence protein B/C

Based on metatranscriptomic data, strikingly high transcription of *pil*A gene (type IV pili assembly protein as designated K02651 and K02650 in KEGG database), the leading gene for the assembly of a conservative type IV pili (Tfp), was noticed in TCF-2, TCF-5, and TCF-12 (Fig. 5). Based on the assumption, genes encoding the beneficial physiochemical features shall be actively transcribed, deciphering the function of the vigorous transcribed Tfp assembly in *Anaerolinales* shall bring useful insight into the gradual dominance of this population within TCF community. The Tfp is the most widespread organs of bacterial attachment [26]. As has been already noted, pili are often involved in facilitating adhesion and colonization in a wide variety of scenarios including: host cells attachment in numerous human pathogens such as *Actinobacillus actinomycetemcomitans* [27]; cellulose binding in *Ruminococcus albus* [28] and biofilm formation on stainless steel in *Pseudomonas aeruginosa* [29]. Moreover, the Tfp in *Geobacter sulfurreducens* are electrical-conductive nanowires involving in direct interspecies electron transfer (DIET) between syntrophic patterns [30].

### Cellular adhesion

As shown in Fig. 5, the conservative Tfp cluster observed in *Anaerolinales* contains a series of genes encoding pilus assembly proteins (*pil*A, CpaB, CpaE, CpaF) and two consecutive Tad proteins (TadB, TadC). The adhesive nature of the Tfp of *Anaerolinales* could be inferred from the occupation and expression of the Tad (tight adhesion) locus which is the essential machinery for the assembly of adhesive pili [31]. The precursor of the major structure component of Tfp is encoded by *pil*A gene. This precursor seems regulatory crucial for effectiveness of Tfp in *Anaerolinales* because the Tfp cluster without proceeding *pil*A genes in TCF-8 and TCF-13 showed no transcriptional activities (Fig. 5). Given TCF-8 and 13′s comparable metabolic capacities to the dominating *Anaerolineales*, such ineffectiveness of Tfp cluster may play a role for their rareness in the community. Additionally, in contrast to the low expression level in carbohydrate metabolism, all the homolog copies of the *pilA* genes in TCF-2, TCF-5, and TCF-12 (respectively encoded 3, 2, and 2 genes annotated as homologs of this enzyme class) got expressed, suggesting the ecological benefits endorsed by pilA expression in *Anaerolinales.*

**Fig. 6:**
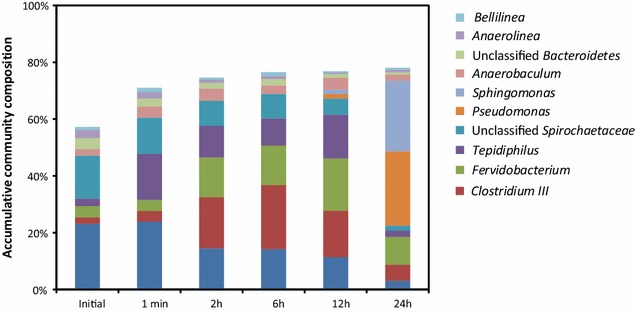
Composition of the bacterial community during attachment. Only prevalent populations taking >1 % of the community are shown

To further investigate the condition of *Anaerolinales* adherence, experiment was conducted to reveal the community change on the surface of filter paper (made of 98 % of microcrystalline cellulose) during hydrolysis. As community profiling based on HTS of 16S rRNA gene amplicons revealed, comparing to the evident accumulation of *Clostridium* and *Fervidobacterium,* populations of *Anaerolinales* (*Anaerolineae and Bellilineae*), though were among the most prevalent populations attached, stayed unchanged in size during the first 12 h of hydrolysis, suggesting its relative incompetence to grow on cellulose surface (Fig. 6). This observation was consistent with the reluctant expression of cellulase genes in the retrieved genomes bins of this lineage. Additionally, increasing bacterial diversity (Additional file 1: Figure S5) in the attached community was induced by the more degradable alpha- and beta-monosaccharides generated at the steady phase of hydrolysis (after 24 h, Figure S5). Remarkable increase of *Sphingomonas* and *Pseudomonas* was observed at this stage, but the paucity of *Anaerolineae* stayed unaffected. Although being commonly regarded as aerobic, strains of *Sphingomonas* and *Pseudomonas* had been reported to be tolerant to anaerobic environment [32–35]. The overgrowth of these two genera in the attached community after 24 h of incubation may be originated from a combination of their ability to utilize beta-linked monosaccharides released from the hydrolysis process [36] as well as their extraordinary ability to grow in biofilm [37, 38]. These results indicated the accumulation of *Anaerolinales* took place other than directly on the surface of cellulose, therefore, we speculate, instead of initiating the attachment on substrate surface, the adhesive feature of *Anaerolineae* enabled by active *pil*A expression might serve as the adhesive matrix for the aggregation of fermentative population in the liquid phase. Since, most anaerobic cellulolytic microorganisms grow optimally on cellulose when attached to the substrate and in at least a few species this adhesion appears to be obligate [36], this surface-free life style of *Anaerolinales* reflected its incompetence in cellulose hydrolysis as disclosed by metatranscriptome. The continuous stirring provided in the enrichment SBR may play the selective role for *Anaerolinales* that microorganisms capable of attaching to each other would benefit from a more efficient exchange of fermentation intermediates and thus proliferate more effectively in competition with other free-ranging anaerobic fermentative counterparts [39]. The advantageous bonding capacity in *Anaerolinales* observed in this study may provide a novel insight into its ubiquity and accumulation in anaerobic digestive systems.

### Syntrophic metabolism

Another interesting function of Tfp is its conductive role for syntrophic DIET. Since the *Anaerolineae* lineage of *Chloroflexi* was considered as semi-syntrophic in anaerobic systems [1] and its interspecies electron transfer (IET) mechanism in mutualistic cooperation with methanogens was yet to be studied, study on the syntrophic machinery and DIET involvement of *Anaerolineae* is indispensable.

Despite the lack of detected transcriptional activities, the shared 926 genes between TCF-8 and TCF-13 (Additional file 1: Figure S2b) revealed a genetic potential of these populations to metabolize ethanol to acetate (Additional file 1: Figure S4), implying their putative role as anaerobic syntrophs. However, these pathways were absent from *Anaerolinales* containing TCF-2, 5, 12, and UNI-1. Additionally, by comparing the transcriptional activities of genes involved in the fundamental steps of syntrophic metabolism as proposed by Sieber et al. [40], relatively weak activities of hydrogenase and formate dehydrogenase suggested the unsteady involvement of H_2_ or formate as the electron carrier for IET in *Anaerolineae* populations (Table 2). Researchers believed genomic co-occurrence of *pil*A and outer membrane cytochromes was prerequisite for DIET to take place in a microbe [41]. Despite the active *pil*A, none of the five curated genome bins and UNI-1 possesses c-type cytochromes (Table 2). As a result, we cannot confirm nor exclude the DIET potential based on the paradox between highly active *pil*A gene and absence of membrane cytochromes in TCF-2, 5, and 12. As a result, consecutive iron supplementation batch tests were designed to verify the DIET potential of the TCF community based on the hypothesis that electric-based syntrophic methanogenesis could be expedited by the dosage of conductive iron-oxide minerals [42]. Fe_2_O_3_ powder was dosed at 20 mM of iron atom [42] to stimulate the electron exchange within the TCF community in three consecutive batches. But batches with iron-oxide supplementation showed no evident advancement on the overall methanogenesis in the short- (1st batch) and long-term (3rd batch) run (Additional file 1: Figure S6a). These batch results indicated that the possibility of DIET phenomenon among microbial populations within the TCF community was rare and thus rejected the initial speculation on DIET involvement of the highly active Tfp in TCF-2, 5, and 12.

**Table 2:** Transcriptional activities of genes potentially involved in interspecies electron transfer for syntrophic metabolism of the five curated *Anaerolineae* genomes

Interspecies electron transfer				
	DIET		H_2_/Formate	
	PilA^a^	CytC^a^	Hyd^a^	FDH^a^
TCF-2	984.5	N/A	0.0	0.3
TCF-5	261.5	N/A	1.6	0.2
TCF-12	1651.6	N/A	0	0
TCF-8	0	N/A	0	0
TCF-13	0	N/A	0	0

MRPKM values were used to evaluate the transcriptional activities of different genes. For *pilA* gene, only the transcription of *pil*A assembly conserved in all the retrieved genomes as shown in Fig. 5 was included

*N/A* the target gene is absent from the curated genomes, *pilA* pilus assembly protein *Flp/PilA,* *CytC* c-type cytochrome, MtrC/OmcA family, *Hyd* hydrogenase, *FDH* formate dehydrogenase

^a^Gene identification is based on the integration of COGs, TIGRFAMs, and KEGG KO orthology as listed in Additional file 1: Table S9

## Conclusion

Coverage-based genome recovery coupled with metatranscriptomic interpretation was used to disclose the advantageous features of *Anaerolineae* populations in anaerobic digestive system based on the five near-complete genomes retrieved from the TCF community. Despite the slight transcription of cellulolytic genes, the prevalence of this population should more likely interrelate with the evident cellular adhesiveness enabled by active transcription of Tfp. Further experiment showed this Tfp structure was functioned as adhesive matrix for cell–cell aggregation other than cell-surface attachment for biofilm initiation nor electron transfer for syntrophic methanogenesis.

## Methods

### Enrichment reactor setup

Anaerobic digestion sludge (ADS) collected from Shek Wu Hui Wastewater Treatment Plant (Hong Kong, SRA, China) were used for the enrichment of thermophilic cellulolytic consortium in a sequential batch reactor (SBR) as described previously [25]. Enriched thermophilic cellulose-fermenting (TCF) sludge was sampled at two different time points (SE: short-term enrichment at 120 days and LE: long-term enrichment at 545 days) during the enrichment.

### Metagenomic binning

#### Metagenomic libraries and Illumina sequencing

Two metagenomic libraries were constructed with genomic DNA respectively extracted from the SE and LE sludge samples. Genomic DNA was extracted from 500 mg dry weight sludge sample with FastDNA^®^ SPIN Kit for Soil (MP Biomedicals, LLC, Illkirch, France). Sequencing of the metagenomic DNA was carried out on the Illumina Hiseq 2000 platform at BGI (Shenzhen, China) by applying the 101 bp paired-end strategy with combined insert lengths of 180 and 800 bp for SE metagenome and sole 180 bp insert for LE metagenome (Additional file 1: Table S2). The resulted PE reads were trimmed for sequencing adaptors before filtering out reads with average phred quality score lower than 20 and ambiguous nucleotide using PRINSEQ [43]. The shotgun metagenomic reads have been deposited into the MG-RAST server for data sharing (see Table S1 for the accession number). SE and LE metagenomes and LE metatranscriptome have been used in our previous studies with focus other than *Anaerolineae* populations [25, 44].

#### De novo assembly and two-dimensional coverage binning

De novo assembly by three popular de novo assemblers, namely MetaVelvet (1.2.01) [45], IDBA_UD (1.1.1) [46], and CLCbio Genomic Workbench 6.0.2 (CLCbio, Denmark), were compared in terms of reads utilization efficiency and length of scaffolds (Additional file 1: Table S9). The most comprehensive IDBA_UD were picked to assemble the SE and LE metagenomes together using a series of kmer 20,40,60,80, and 100. Two metagenomes were assembled together to facilitate generation of long scaffolds. Only scaffolds longer than 1 kb were kept for subsequent genomic binning analysis.

Based on the assumption that scaffolds belonging to the same genome (strain) should share similar coverage across different metagenomes, scaffolds of targeted *Anaerolineae* genome bins were recruited from the two-dimensional coverage plot using R scripts [13]. Divergent coverage of *Chloroflexi* populations were provided by metagenomic libraries of thermophilic cellulolytic sludge sampled from the same reactor but at two different times (SE at 120 days and LE at 545 days). The coverage sets of scaffolds were obtained by independently mapping PE reads in the SE and LE metagenomes against scaffolds assembled, using Bowtie 1.0.1 [47] allowing two mismatches over the entire read length (bowtie option: −v 2 −m 200) [20]. Coverage of a scaffold was calculated as the total base pairs of mapped read divided by its length. After that, the scaffolds were binned based on the clustering of coverage and phylum assignment. To minimize the potential contamination, another genomic signature, tetra-nucleotide frequency (TNF), was used to refine the bins at euclidean distance cutoff of 0.1 [20]. Finally, PE-tracking tools from the mm genome package [13] was used to reinforce the scaffolding by retrieving genes initially excluded, for example, genes showing deviate coverage caused by multiple copies.

At the same time, community composition was assessed by identifying 16S rRNA sequences in metagenomes. The unassembled illumina reads were searched against Silva SSU 115 database [48] with BLASTN [49] using evalue cutoff of 1E−20. The tabular BLAST results were parsed at phylum level with MEGAN4 [50] using the lowest common ancestor algorithm.

### Genome completeness, contamination, and abundance in metagenomes

The HMM of 107 essential single-copy genes (ESCGs) (Additional file 1: Table S4), defined as the single-copy genes conserved in 95 % of all bacteria [51], were used as pan-genome to indicate the completeness and potential contamination of the genome bins. The completeness of a draft genome was measured by the percentage of identified ESCGs out of the total 107 ESCGs, while the contamination was determined as dividing duplicated ESCGs by the number of ESCGs identified in the draft genome. To double check our estimation on completeness and purity of a draft genome, a set of 35 orthologous groups (COGs) [21] (Additional file 1: Table S6) were used as alternative markers. The relative abundance of each curated genome bin in a metagenome was calculated as the number of reads mapped in percentage of the total number of reads in a metagenome. ANI is calculated with similarity cutoff of 60 % [23], while DDH was in silico estimated by GGDC [52].

### Reconstruction of *16S rRNA* genes

Complete *16S rRNA* gene of the genome bins TCF-2, 5, and 12 were determined by IMG 4.0 genome annotation pipeline [53] and double confirmed by EMIRGE [54]. EMIRGE was used as a complementary approach to reconstruct 16S rRNA genes from the shotgun libraries with 80 iterations. Uchime [55] was used to filter the possible chimera formed in EMIRGE before comparing the reconstructed 16S rRNA gene to that of the curated genome bins. The incomplete prediction of *16S rRNA* gene in TCF-13 (258 bp) was manually extended based on its nearly identical BLAST match (similarity higher than 99 % over 258 bp) to a 16S rRNA sequence in Silva SSU database (version 11.5).

### Phylogenetic analysis of draft genomes

In order to determine the phylogenetic position of draft genomes obtained here, neighbor-joining tree of *Anaerolineae* was built using MEGA5 [56] with maximum-likelihood method and bootstrap value of 1000. A phylogenetic tree was constructed using (1) 16S rRNA sequences of the draft genomes, (2) *16S rRNA* gene of *A.thermophila* UNI-1, (3) 16S rRNA gene of ten isolated strains and high-quality 16S clones collected from Silva SSU database.

To determine the phylogenetic affiliation of TCF-8 whose *16S rRNA* gene is too short for reliable alignment, genome tree was constructed from a concatenated alignment of 35 protein-coding ESCGs shared in single-copy manner among the five curated genomes and twenty-two finished genomes of *Chloroflexi* in IMG 4.0. A maximum-likelihood tree was created using phyml 3.1 [57] using default setting for amino acids with 100 bootstraps based on MUSLE [58] alignments.

### Functional and transcription analysis

#### Functional annotation of the *Anaerolineae* genomes

The five near-complete genomic bins retrieved from the TCF community were submitted to IMG annotation pipeline for ORF calling as well as functional annotation (The IMG genome ID of each bin was listed in Table 1). IMG annotation on Pfam, KEGG, and COG databases were compared against that of twenty-two finished genomes of *Chloroflexi* to reveal metabolism styles. Given the unavailability of syntrophic pathways in a single database, identification of key genes involved in the syntrophic process in the present study was based on the integration of COG, PfamA, TIGRFAMs, as well as KEGG KO annotation (The identifier of syntrophic metabolism related genes used in this study are listed in Additional file 1: Table S10).

### Metatranscriptomic sequencing and expression quantification

Total RNA of the LE sludge sample was extracted and then sequenced following the protocol described previously [25]. Transcriptional activities of genes in each draft genome were investigated in the same manner as previously established [25]. Briefly, the concept of MRPKM, defined as the ratio of RPKM-RNA to RPKM-DNA, was used to evaluate the transcriptional activity of genes in metatranscriptome. RPKM-DNA and RPKM-RNA was respectively calculated from metagenome and metatranscriptome of LE sample using RSEM [59] based on Bowtie 1.0.1 alignment allowing two mismatches over the entire read length.

### Iron-oxide supplementation batch tests

#### Reactor setup

50 ml sludge collecting from the enrichment batch at the peak of a SBR cycle was added as seed sludge to batch test with working volume of 100 ml. Medium solution was prepared following previous protocol [60]. Microcrystalline cellulose (50 µm in diameter, Sigma, USA) was dosed at concentration of 2.5 g/l, while Fe_2_O_3_ powder (≥99.995 % trace metals basis, Aldrich, USA) was supplemented to stock solution to give the final concentration of 20 mM as Fe atom [42]. Nitrogen was used to purge out the air inside the serum bottle to ensure anaerobic environment. Batch tests were carried out in 55 °C water bath with continuous stirring at 120 rpm. For each batch test, the cellulose substrate was 2.5 g/l and the initial pH was controlled at around 7.5. Three consecutive batches were conducted to investigate the effect of iron supplementation in the short- and long-term run. Each batch was suspended when biogas generation ceased for all the reactors. The results represented were average value of duplicated tests.

#### Gas and volatile fatty acids analysis

Gas volume was monitored by a glass syringe. Gas content, including hydrogen, methane, and carbon dioxide, were determined using gas chromatograph (GC-TCD) following configuration described previously [60]. The composition in the liquid phase including volatile fatty acids and alcohols, were measured using a second GC-FID [60].

### Attachment experiment and community profiling by high-throughput sequencing

Slices of filter paper (Whatman, 98 % microcyrstalline cellulose) were dipped into the thermophilic SBR (run for 716 days) for a certain time (1 min and 2, 6, 12, 24, 30, and 32 h) to accumulate microbes that attached to cellulose surface. Biological replicates were sampled at 12 h (Additional file 1: Figure S7). Filter paper dipped for 1 min was used to represent the community binded to filter paper by physical adsorption. The initial community before experiment was also sampled. Filter paper after dipping was washed with DI waster to kept microbial populations steadily attached to the surface [20]. The sampled filter paper was cut in half, with each half respectively used for DNA extraction (with the same protocol for metagenomic DNA extraction) and weight measuring. Dry weight lost was used to evaluate the hydrolysis efficiency (Additional file 1: Figure S8). Universal primers for V4 FLX forward primer (“AYTGGGYDTAAAGNG”) and reverse primers (“TACNVGGGTATCTAATCC”, “TACCRGGGTHTCTAATCC”, TACCAGAGTATCTAATTC”, “CTACDSRGGTMTCTAATC”) targeting the V4 region of *16S rRNA* gene were used to amplify genetic amplicons for community profiling using Roche FLX 454 high-throughput sequencing (HTS) at BGI (Shenzhen, China). Slice of unused filter paper was also subject to the same DNA extraction and *16S rRNA* gene amplification to testify primer specificity towards microbial populations (Additional file 1: Figure S9).

Quality filtering and community analysis of the 454 reads was conducted following protocol previous reported [61]. Briefly, the raw reads were demultiplexed, quality trimmed, aligned, and finally checked with ChimeraSlayer to remove chimeric sequences by standard procedure in Mothur [62]. The post quality filtering reads (Additional file 1: Table S11) were clustered into operational taxonomic units (OTUs) equivalent to genus level (0.97 similarity) by open OTU algorithm adopted in QIIME platform [63]. Taxonomy of each OTU was assigned by RDP Classifier [64] using confidence threshold of 50 % which provides a trade-off between adequate classification accuracy and maximizing the percentage of classifiable sequences [65]. Discussion on the community composition only focus on the prevalent populations taking >1 % of the bacterial community.

## Additional file

10.1186/s13068-016-0524-z Metagenomic and metatranscriptomic libraries of the thermophilic cellulose-degrading consortium.** Table S2**. Statistics on the scaffolds obtained from de novo assembly by IDBA-UD using EE and LE metagenomes together.** Table S3**. Relative abundance of each genome bin and genome completeness and contamination potential estimated based on 107 ESCGs.** Table S4**. List of 107 HMM of ESCGs conserved in 95 of bacteria and their representation in the five Anaerolinea draft genomes for completeness estimation.** Table S5**. Estimation of the validate range of genome completeness and ESCG redundancy by twenty finished genomes of Chloroflexi.** Table S6**. List of 35 COG marker and their representation in the five Anaerolineae draft genomes for completeness confirmation.** Table S7**. Summary of other draft genome bins retrieved from the metagenome.** Table S8**. Metabolic characteristics of isolated strains of Anaerolineae.** Table S9**. Comparison of assembly by three different de novo assemblers. Only the EE metagenome were assembled for comparison.** Table S10**. Functional orthologues of genes putatively involve in electron transfer for syntrophic metabolism.** Table S11**. Statistic of the post-QC HTS reads of 16S rRNA gene amplicons of the attachment samples.** Figure S1**. Community structure of the TCF consortium showing the accumulation of Chloroflexi during long-term run of the enrichment SBR.** Figure S2**. (a): Table showing the in-silico DNA-DNA hybridization values (DDH) (upper diagonal) and Average Nucleotide Identity (ANI) (lower diagonal) among five curated genomes retrieved and A. thermophila UNI-1 and C. aerophila DSM14535. Genomes in the table are ordered according to the phylogenetic relationship represented by the concatenated tree based on 35 shared ESCGs (to the left of the table). The number of aligned fragments used for ANI calculation is shown in bracket under the ANI value. (b) Venn diagram showing the number of shared and unique genes between TCF-8 and TCF-13 based on KEGG orthology annotation. (c) Venn diagram showing the number of shared and unique genes among TCF-2, 5, 12 and A. thermophila UNI-1 based on KEGG orthology annotation.** Figure S3**. Hierarchy clustering of members of Chloroflexi based on Euclidean distance of COGs annotation of 32 available genomes of Chloroflexi phylum and five curated genomes retrieved. Finished genomes [F], permanent draft genomes [P] and draft genomes [D] were all considered to insure comprehensive functional comparison.** Figure S4**. Transcriptional activities of genes involve in the Glycolysis pathway (partially shown) in the five curated bins and A. thermophila UNI-1. Filled blocks in the bottom and top role respectively represent genes encoded and expressed in the corresponding genomes Blocks are filled with the same position and color as the corresponding genomes in the legend.** Figure S5**. Rarefaction analysis of the community attached to cellulose surface. Top and bottom sub-tables respectively represent the rarefaction curve of Archaea (top) and Bacterial (bottom) community.** Figure S6**. Comparison of methane (CH4) and major VFAs (acetic acid and propanoic acid) generation between Iron-supplemented (in form of Fe2O3) and control in consecutive batch tests. B1: the first batch; B3: the last consecutive batch.** Figure S7**. Bacterial (left) and Archaea (right) community correlation between biological replicates sampled at twelve hours. Only prevalent populations taking >1 % of the community are considered in correlation test.** Figure S8**. Hydrolysis during attachment. The error bar represents the deviations between biological replicates sampled at twelve hours.** Figure S9**. Electrophoretogram of the 16S rRNA genes amplicons used for high-throughput sequencing. Blank represents the band of filter paper. PCR products of 30 and 48 hours was not used in the sequencing. Takara DL2000 was used as marker.** Figure S10**. Composition of Archaea community during attachment. Only prevalent population taking > 1 % in the community are shown in the figure.

## Abbreviations

TCF: thermophilic cellulose fermenting
CAG: carbohydrate-active gene
UASB: upflow anaerobic sludge blanket reactor
UNI-1: *Anaerolinea thermophila* UNI-1
Tfp: type VI pili
NGS: next generation sequencing
TNF: tetra-nucleotide frequency
SE: short-term enrichment
LE: long-term enrichment
ESCG: essential single-copy genes
ANI: average nucleotide identity
DDH: DNA–DNA hybridization value
CMC: cellulase M cluster
CBM: carbohydrate-binding modules
ADS: anaerobic digestion sludge
SBR: sequential batch reactor
IET: interspecies electron transfer
DIET: direct interspecies electron transfer
HTS: high-throughput sequencing
OTU: operational taxonomic units
