# Supplementary material for: Cellular adhesiveness and cellulolytic capacity in Anaerolineae revealed by omics-based genome interpretation
Source: Biotechnol Biofuels. 2016 May 23;9:111. doi: 10.1186/s13068-016-0524-z (PMC4877987; doi:10.1186/s13068-016-0524-z)
Supplement: Supplementary file 1 — 10.1186/s13068-016-0524-z Metagenomic and metatranscriptomic libraries of the thermophilic cellulose-degrading consortium. Table S2. Statistics on the scaffolds obtained from de novo assembly by IDBA-UD using EE and LE metagenomes together. Table S3. Relative abundance of each genome bin and genome completeness and contamination potential estimated based on 107 ESCGs. Table S4. List of 107 HMM of ESCGs conserved in 95 of bacteria and their representation in the five Anaerolinea draft genomes for completeness estimation. Table S5. Estimation of the validate range of genome completeness and ESCG redundancy by twenty finished genomes of Chloroflexi. Table S6. List of 35 COG marker and their representation in the five Anaerolineae draft genomes for completeness confirmation. Table S7. Summary of other draft genome bins retrieved from the metagenome. Table S8. Metabolic characteristics of isolated strains of Anaerolineae. Table S9. Comparison of assembly by three different de novo assemblers. Only the EE metagenome were assembled for comparison. Table S10. Functional orthologues of genes putatively involve in electron transfer for syntrophic metabolism. Table S11. Statistic of the post-QC HTS reads of 16S rRNA gene amplicons of the attachment samples. Figure S1. Community structure of the TCF consortium showing the accumulation of Chloroflexi during long-term run of the enrichment SBR. Figure S2. (a): Table showing the in-silico DNA-DNA hybridization values (DDH) (upper diagonal) and Average Nucleotide Identity (ANI) (lower diagonal) among five curated genomes retrieved and A. thermophila UNI-1 and C. aerophila DSM14535. Genomes in the table are ordered according to the phylogenetic relationship represented by the concatenated tree based on 35 shared ESCGs (to the left of the table). The number of aligned fragments used for ANI calculation is shown in bracket under the ANI value. (b) Venn diagram showing the number of shared and unique genes between TCF-8 and TCF-13 base [file 13068_2016_524_MOESM1_ESM.doc]

**Cellular adhesiveness and cellulolytic capacity in *Anaerolineae* revealed by Omics-based genome interpretation**

Yu Xia ^1^, Yubo Wang ^1^, Yi Wang ^2a^, Francis Y.L. Chin ^2^, Tong Zhang ^1^#

1. Metagenomic and metatranscriptomic libraries of the thermophilic cellulose-degrading consortium

| Library category | Sampling time | Library insert length | Data size after QC | Read number after QC | Average read length after QC | MG-RAST Accession number |
| --- | --- | --- | --- | --- | --- | --- |
| Metagenomic library | 120days | 180 bp | 0.9 G | 9,706,404 | 100 bp | 4480034 4503057 |
|  |  | 800 bp | 0.8 G | 9,796,496 | 88 bp | 4503057 |
|  | 549days | 180 bp | 5.0 G | 50,255,458 | 100 bp | 4503055 4503056 |
| Metatranscriptomic library | 549days | 180 bp | 6.0 G | 59,777,242 | 100 bp | 4506806 4506808 4506807 4506809 |

1. Statistics on the scaffolds obtained from de novo assembly by IDBA-UD using SE and LE metagenomes together

|  | >300 bp | >1000 bp | | > 5000 bp | >10000 bp |
| --- | --- | --- | --- | --- | --- |
| total size, Mb | 157 | 119 | | 84 | 75 |
| number of contigs | 395,330 | 19,653 | | 4,105 | 2,177 |
| N50,bp | 8260 | 19,859 | | 37,484 | 48,884 |
| N90,bp | 466 | 1,825 | | 7,801 | 14,013 |
| Reads utilization percentage | | | 86.7% | | |
| Longest scaffold | | | 640,223 bp | | |

1. Relative abundance of each genome bin and genome completeness and contamination potential estimated based on 107 ESCGs.

| Genome name | Genome size (Mb) | NO. of scaffolds | GC (%) | Genome completeness (%) in 107 ESCGs | Genome redundancy (%) in 107 ESCGs | SE relative abundance^1)^ (%) | LE relative abundance^1)^ (%) |
| --- | --- | --- | --- | --- | --- | --- | --- |
| TCF-2 | 3.8 | 55 | 54.4 | 99.1 | 3.8 | 14.1 | 11.7 |
| TCF-5 | 3.0 | 27 | 55.4 | 98.1 | 1.9 | 0.2 | 7.3 |
| TCF-8 | 4.1 | 51 | 63.6 | 99.1 | 2.8 | 4.7 | 0.8 |
| TCF-12 | 3.7 | 69 | 53.4 | 100.0 | 3.7 | 0.0 | 2.2 |
| TCF-13 | 4.0 | 153 | 64.9 | 100.0 | 3.7 | 0.2 | 1.9 |
| TCF-14 | 3.1 | 48 | 54.0 | 76.6 | 2.4 | 0.0 | 1.0 |

^1)^ The relative abundance of each draft genome was estimated as the number of reads mapped to the draft genome in percentage of the total number of reads in the metagenome.

1. List of 107 HMM of ESCGs conserved in 95 of bacteria (Dupont et al., 2012) and their representation in the five Anaerolinea draft genomes for completeness estimation

| Accession | Name | TCF-2 | TCF-5 | TCF-8 | TCF-12 | TCF-13 |
| --- | --- | --- | --- | --- | --- | --- |
| TIGR00344 | alanine--tRNA ligase | 1 | 1 | 1 | 1 | 1 |
| PF00750 | arginine--tRNA ligase | 1 | 1 | 1 | 1 | 1 |
| TIGR00459 | aspartate--tRNA ligase | 1 | 0 | 1 | 0 | 1 |
| TIGR02350 | chaperone protein DnaK | 2 | 2 | 1 | 2 | 1 |
| TIGR00362 | chromosomal replication initiator protein DnaA | 1 | 1 | 1 | 1 | 0 |
| PF01025 | co-chaperone GrpE | 1 | 1 | 1 | 2 | 1 |
| TIGR00337 | CTP synthase | 1 | 1 | 1 | 1 | 1 |
| TIGR00435 | cysteine--tRNA ligase | 1 | 1 | 1 | 1 | 1 |
| TIGR00152 | dephospho-CoA kinase | 1 | 1 | 1 | 1 | 1 |
| TIGR01063 | DNA gyrase, A subunit | 2 | 1 | 1 | 2 | 2 |
| TIGR01059 | DNA gyrase, B subunit | 1 | 1 | 1 | 1 | 1 |
| TIGR00575 | DNA ligase, NAD-dependent | 1 | 1 | 1 | 1 | 1 |
| TIGR00663 | DNA polymerase III, beta subunit | 2 | 1 | 1 | 1 | 1 |
| TIGR02397 | DNA polymerase III, subunit gamma and tau | 1 | 1 | 1 | 1 | 1 |
| TIGR01391 | DNA primase | 1 | 1 | 1 | 1 | 1 |
| TIGR02027 | DNA-directed RNA polymerase, alpha subunit | 1 | 1 | 1 | 1 | 1 |
| TIGR02013 | DNA-directed RNA polymerase, beta subunit | 1 | 1 | 1 | 1 | 1 |
| TIGR02386 | DNA-directed RNA polymerase, beta' subunit | 1 | 1 | 1 | 1 | 1 |
| TIGR01393 | elongation factor 4 | 1 | 1 | 1 | 1 | 1 |
| TIGR00631 | excinuclease ABC subunit B | 1 | 1 | 1 | 1 | 1 |
| TIGR00388/  TIGR00389 | glycine--tRNA ligase, alpha subunit | 1 | 1 | 1 | 1 | 1 |
| TIGR00436 | GTP-binding protein Era | 2 | 2 | 2 | 2 | 2 |
| TIGR00092 | GTP-binding protein YchF | 1 | 1 | 1 | 1 | 1 |
| TIGR03263 | guanylate kinase | 1 | 1 | 1 | 1 | 0 |
| TIGR00442 | histidine--tRNA ligase | 1 | 1 | 1 | 1 | 1 |
| TIGR00392 | isoleucine--tRNA ligase | 1 | 1 | 1 | 1 | 1 |
| TIGR00396 | leucine--tRNA ligase | 1 | 1 | 1 | 1 | 1 |
| TIGR00460 | methionyl-tRNA formyltransferase | 1 | 1 | 1 | 1 | 1 |
| PF01795 | MraW methylase family | 1 | 1 | 0 | 1 | 1 |
| TIGR02729 | Obg family GTPase CgtA | 1 | 1 | 0 | 1 | 1 |
| TIGR00019 | peptide chain release factor 1 | 1 | 1 | 1 | 1 | 0 |
| TIGR00468 | phenylalanine--tRNA ligase, alpha subunit | 1 | 1 | 0 | 1 | 0 |
| TIGR00471/  TIGR00472 | phenylalanine--tRNA ligase, beta subunit | 1 | 1 | 1 | 1 | 1 |

| PF00162 | phosphoglycerate kinase | 1 | 1 | 1 | 1 | 1 |
| --- | --- | --- | --- | --- | --- | --- |
| TIGR00963 | preprotein translocase, SecA subunit | 1 | 1 | 1 | 1 | 1 |
| TIGR00964 | preprotein translocase, SecE subunit | 1 | 1 | 1 | 1 | 1 |
| TIGR00810 | preprotein translocase, SecG subunit | 1 | 1 | 1 | 1 | 1 |
| TIGR00967 | preprotein translocase, SecY subunit | 1 | 1 | 1 | 1 | 1 |
| TIGR00043 | probable rRNA maturation factor YbeY | 1 | 1 | 1 | 1 | 1 |
| TIGR00408 | proline--tRNA ligase | 1 | 1 | 1 | 1 | 1 |
| TIGR00409 | proline--tRNA ligase | 0 | 0 | 1 | 1 | 1 |
| TIGR02012 | protein RecA | 1 | 1 | 2 | 1 | 2 |
| TIGR02191 | ribonuclease III | 1 | 1 | 1 | 1 | 1 |
| TIGR01169 | ribosomal protein L1 | 1 | 1 | 1 | 1 | 1 |
| PF00466 | ribosomal protein L10 | 1 | 1 | 1 | 1 | 1 |
| TIGR01632 | ribosomal protein L11 | 1 | 1 | 1 | 1 | 1 |
| TIGR01066 | ribosomal protein L13 | 1 | 1 | 1 | 1 | 1 |
| TIGR01067 | ribosomal protein L14 | 1 | 1 | 1 | 1 | 1 |
| TIGR01071 | ribosomal protein L15 | 1 | 1 | 1 | 1 | 1 |
| TIGR01164 | ribosomal protein L16 | 1 | 1 | 1 | 1 | 1 |
| TIGR00059 | ribosomal protein L17 | 1 | 1 | 1 | 1 | 1 |
| TIGR00060 | ribosomal protein L18 | 1 | 1 | 1 | 1 | 1 |
| TIGR01024 | ribosomal protein L19 | 1 | 1 | 0 | 1 | 1 |
| TIGR01171 | ribosomal protein L2 | 1 | 1 | 1 | 1 | 1 |
| TIGR01032 | ribosomal protein L20 | 1 | 1 | 1 | 1 | 1 |
| TIGR00061 | ribosomal protein L21 | 0 | 1 | 1 | 1 | 1 |
| TIGR01044 | ribosomal protein L22 | 1 | 1 | 1 | 1 | 1 |
| PF00276 | ribosomal protein L23 | 1 | 1 | 1 | 1 | 1 |
| TIGR01079 | ribosomal protein L24 | 1 | 1 | 1 | 1 | 1 |
| TIGR00062 | ribosomal protein L27 | 0 | 1 | 1 | 1 | 1 |
| TIGR00009 | ribosomal protein L28 | 0 | 0 | 1 | 0 | 1 |
| TIGR00012 | ribosomal protein L29 | 1 | 1 | 1 | 1 | 1 |
| PF00297 | ribosomal protein L3 | 1 | 1 | 1 | 1 | 1 |
| TIGR01031 | ribosomal protein L32 | 1 | 1 | 1 | 1 | 1 |
| TIGR01030 | ribosomal protein L34 | 1 | 1 | 1 | 1 | 1 |
| TIGR00001 | ribosomal protein L35 | 0 | 0 | 1 | 0 | 1 |
| PF00573 | ribosomal protein L4/L1 family | 1 | 1 | 1 | 1 | 1 |
| PF00281 | ribosomal protein L5 | 1 | 1 | 1 | 1 | 1 |
| PF00347 | ribosomal protein L6 | 1 | 1 | 1 | 1 | 1 |
| TIGR00855 | ribosomal protein L7/L12 | 1 | 1 | 1 | 1 | 1 |
| TIGR00158 | ribosomal protein L9 | 1 | 1 | 1 | 1 | 1 |
| TIGR01049 | ribosomal protein S10 | 1 | 1 | 1 | 1 | 1 |
| PF00411 | ribosomal protein S11 | 1 | 1 | 1 | 1 | 1 |
| TIGR00981 | ribosomal protein S12 | 1 | 1 | 1 | 1 | 1 |
| PF00416 | ribosomal protein S13p/S18e | 1 | 1 | 2 | 1 | 1 |
| TIGR00952 | ribosomal protein S15 | 1 | 1 | 1 | 1 | 1 |
| TIGR00002 | ribosomal protein S16 | 1 | 1 | 1 | 1 | 1 |
| PF00366 | ribosomal protein S17 | 1 | 1 | 1 | 1 | 1 |
| TIGR00165 | ribosomal protein S18 | 1 | 1 | 1 | 1 | 1 |
| TIGR01050 | ribosomal protein S19 | 1 | 1 | 1 | 1 | 1 |
| TIGR01011 | ribosomal protein S2 | 1 | 1 | 1 | 1 | 1 |
| TIGR00029 | ribosomal protein S20 | 1 | 1 | 1 | 1 | 1 |
| TIGR01009 | ribosomal protein S3 | 1 | 1 | 1 | 1 | 1 |
| TIGR01017 | ribosomal protein S4 | 1 | 1 | 1 | 1 | 1 |
| TIGR01021 | ribosomal protein S5 | 1 | 1 | 1 | 1 | 1 |
| TIGR00166 | ribosomal protein S6 | 1 | 1 | 1 | 1 | 1 |
| TIGR01029 | ribosomal protein S7 | 1 | 1 | 1 | 1 | 1 |
| PF00410 | ribosomal protein S8 | 1 | 1 | 1 | 1 | 1 |
| PF00380 | ribosomal protein S9 | 1 | 1 | 1 | 1 | 1 |
| TIGR00496 | ribosome recycling factor | 1 | 1 | 1 | 1 | 1 |
| TIGR03594 | ribosome-associated GTPase EngA | 1 | 1 | 1 | 1 | 1 |
| TIGR00082 | ribosome-binding factor A | 1 | 1 | 1 | 1 | 1 |
| TIGR00414 | serine--tRNA ligase | 1 | 1 | 1 | 1 | 1 |
| TIGR00959 | signal recognition particle protein | 1 | 1 | 1 | 1 | 1 |
| TIGR00064 | signal recognition particle-docking protein FtsY | 1 | 1 | 1 | 1 | 1 |
| TIGR00086 | SsrA-binding protein | 1 | 1 | 1 | 1 | 1 |
| TIGR00418 | threonine--tRNA ligase | 1 | 1 | 1 | 1 | 1 |
| TIGR01953 | transcription termination factor NusA | 1 | 1 | 1 | 1 | 1 |
| TIGR00922 | transcription termination/antitermination factor NusG | 1 | 1 | 1 | 1 | 1 |
| TIGR00116 | translation elongation factor Ts | 1 | 1 | 1 | 1 | 1 |
| TIGR00487 | translation initiation factor IF-2 | 1 | 1 | 1 | 1 | 1 |
| TIGR00168 | translation initiation factor IF-3 | 1 | 1 | 1 | 1 | 1 |
| TIGR00115 | trigger factor | 1 | 1 | 1 | 1 | 1 |
| TIGR00420 | tRNA(5-methylaminomethyl-2-thiouridylate)-methyltransferase | 1 | 1 | 1 | 1 | 2 |
| TIGR02432 | tRNA(Ile)-lysidine synthetase | 1 | 1 | 1 | 1 | 1 |
| TIGR00234 | tyrosine--tRNA ligase | 1 | 1 | 1 | 1 | 1 |
| TIGR00422 | valine--tRNA ligase | 1 | 1 | 1 | 1 | 1 |
| Number of total ESCGs in each genome | | 106 | 105 | 106 | 107 | 107 |
| Number of unique ESCGs in each genome | | 102 | 103 | 103 | 104 | 103 |
| Number of unique ESCGs in the *Chloroflexi* | | 103 | 103 | 103 | 103 | 103 |
| Genome completeness (%) in total 107 ESCGs | | 99.1 | 98.1 | 99.1 | 100 | 100 |
| Genome redundancy (%) in total 107 ESCGs | | 3.8 | 1.9 | 2.8 | 3.7 | 3.7 |

1. Estimation of the validate range of genome completeness and ESCG redundancy by twenty finished genomes of Chloroflexi

| Genome name | Genome completeness (%) in 107 ESCG | Genome redundancy (%) in 107 ESCGs |
| --- | --- | --- |
| *Anaerolinea thermophila* UNI-1 | 99.1 | 2.8 |
| *Caldilinea aerophila* DSM14535 | 97.2 | 1.9 |
| *Chloroflexus aggregans* DSM9485 | 100.0 | 4.7 |
| *Chloroflexus aurantiacus* J-10-fl | 100.0 | 4.7 |
| *Chloroflexus* sp Y396-1 | 100.0 | 4.7 |
| *Chloroflexus* sp. Y400-fl | 100.0 | 4.7 |
| *Dehalococcoides ethenogenes* 195 | 96.3 | 1.0 |
| *Dehalococcoides mccartyi* BAV1 | 96.3 | 1.0 |
| *Dehalococcoides mccartyi* BTF08 | 96.3 | 1.0 |
| *Dehalococcoides mccartyi* CBDB1 | 96.3 | 1.9 |
| *Dehalococcoides mccartyi* DCMB5 | 96.3 | 1.0 |
| *Dehalococcoides mccartyi* GT | 96.3 | 1.0 |
| *Dehalococcoides mccartyi* GY50 | 96.3 | 1.0 |
| *Dehalococcoides mccartyi* VS | 96.3 | 1.0 |
| *Dehalogenimonas lykanthroporepellens* BL-DC-9 | 96.3 | 1.0 |
| *Herpetosiphon aurantiacus* DSM785 | 99.1 | 3.8 |
| *Roseiflexus castenholzii* DSM13941 | 99.1 | 4.7 |
| *Roseiflexus* sp. RS-1 | 99.1 | 4.7 |
| *Sphaerobacter thermophiles* DSM20745 | 98.1 | 4.8 |
| *Thermomicrobium roseum* DSM5159 | 97.2 | 1.9 |

1. *List of 35 COG marker [21] and their representation in the five Anaerolineae draft genomes for completeness confirmation*

| COG Marker | COG Name | TCF-2 | TCF-5 | TCF-8 | TCF-12 | TCF-13 |
| --- | --- | --- | --- | --- | --- | --- |
| COG0012 | Predicted GTPase, probable translation factor | 1 | 1 | 1 | 1 | 1 |
| COG0016 | Phenylalanyl-tRNA synthetase alpha subunit | 1 | 1 | 1 | 1 | 0 |
| COG0048 | Ribosomal protein S12 | 1 | 1 | 1 | 1 | 1 |
| COG0049 | Ribosomal protein S7 | 1 | 1 | 1 | 1 | 1 |
| COG0052 | Ribosomal protein S2 | 1 | 1 | 1 | 1 | 1 |
| COG0080 | Ribosomal protein L11 | 1 | 1 | 1 | 1 | 1 |
| COG0081 | Ribosomal protein L1 | 1 | 1 | 1 | 1 | 1 |
| COG0085 | DNA-directed RNA polymerase, beta subunit/140 kD subunit | 1 | 1 | 1 | 1 | 1 |
| COG0087 | Ribosomal protein L3 | 1 | 1 | 1 | 1 | 1 |
| COG0088 | Ribosomal protein L4 | 1 | 1 | 1 | 1 | 1 |
| COG0090 | Ribosomal protein L2 | 1 | 1 | 1 | 1 | 1 |
| COG0091 | Ribosomal protein L22 | 1 | 1 | 1 | 1 | 1 |
| COG0092 | Ribosomal protein S3 | 1 | 1 | 1 | 1 | 1 |
| COG0093 | Ribosomal protein L14 | 1 | 1 | 1 | 1 | 1 |
| COG0094 | Ribosomal protein L5 | 1 | 1 | 1 | 1 | 1 |
| COG0096 | Ribosomal protein S8 | 1 | 1 | 1 | 1 | 1 |
| COG0097 | Ribosomal protein L6P/L9E | 1 | 1 | 1 | 1 | 1 |
| COG0098 | Ribosomal protein S5 | 1 | 1 | 1 | 1 | 1 |
| COG0099 | Ribosomal protein S13 | 1 | 1 | 1 | 1 | 1 |
| COG0100 | Ribosomal protein S11 | 1 | 1 | 1 | 1 | 1 |
| COG0102 | Ribosomal protein L13 | 1 | 1 | 1 | 1 | 1 |
| COG0103 | Ribosomal protein S9 | 1 | 1 | 1 | 1 | 1 |
| COG0124 | Histidyl-tRNA synthetase | 1 | 1 | 1 | 1 | 1 |
| COG0184 | Ribosomal protein S15P/S13E | 1 | 1 | 1 | 2 | 1 |
| COG0185 | Ribosomal protein S19 | 1 | 1 | 1 | 1 | 1 |
| COG0186 | Ribosomal protein S17 | 1 | 1 | 1 | 1 | 1 |
| COG0197 | Ribosomal protein L16/L10E | 1 | 1 | 1 | 1 | 1 |
| COG0200 | Ribosomal protein L15 | 1 | 1 | 1 | 1 | 1 |
| COG0201 | Preprotein translocase subunit SecY | 1 | 1 | 1 | 1 | 1 |
| COG0256 | Ribosomal protein L18 | 1 | 1 | 1 | 1 | 1 |
| COG0495 | Leucyl-tRNA synthetase | 1 | 1 | 1 | 1 | 2 |
| COG0522 | Ribosomal protein S4 and related proteins | 1 | 1 | 1 | 1 | 1 |
| COG0525 | Valyl-tRNA synthetase | 1 | 1 | 1 | 1 | 1 |
| COG0533 | Metal-dependent proteases with possible chaperone activity | 0 | 1 | 1 | 1 | 1 |
| COG0541 | Signal recognition particle GTPase | 1 | 1 | 1 | 1 | 1 |
| COG marker represented in the genome | | 34 | 35 | 35 | 35 | 34 |
| Estimated genome completeness (%) | | 97 | 100 | 100 | 100 | 97 |
| Estimated genome contamination (%) | | 0 | 0 | 0 | 3 | 3 |

1. Summary of other draft genome bins retrieved from the metagenome

| Genome | Genome size, Mb | scaffolds | GC % | Completeness^1)^ | Redundancy^1)^ | SE^2)^ | LE^2)^ | IMG accession |
| --- | --- | --- | --- | --- | --- | --- | --- | --- |
| 1_Firmicutes | 4.1 | 172 | 35.6 | 100.9% | 2.8% | 46.4% | 6.9% | 2588253526 |
| 3_Bacteroidetes | 2.9 | 137 | 42.7 | 93.5% | 0.0% | 4.1% | 12.0% | 2588253525 |
| 4_Euryarchaeota | 1.5 | 14 | 48.4 | 100.0% | 2.9% | 1.0% | 3.7% | 2588253527 |
| 6_Euryarchaeota | 1.5 | 76 | 50.2 | 88.6% | 1.6% | 5.8% | 0.8% | 2622736600 |
| 7_Euryarchaeota | 2.9 | 34 | 41.2 | 100.0% | 2.9% | 3.0% | 1.0% | 2588253528 |
| 9_Bacteroidetes | 3.1 | 192 | 28.6 | 93.5% | 2.0% | 1.2% | 1.0% | 2588253530 |
| 10_Synergistetes | 1.4 | 267 | 47.6 | 88.8% | 3.2% | 0.9% | 0.9% | 2622736601 |
| 11_Spirochaetes | 2.3 | 132 | 57.2 | 95.3% | 2.0% | 1.1% | 1.5% | 2588253529 |
| 14_Chloroflexi | 3.1 | 48 | 54 | 76.6% | 2.4% | 0.0% | 1.0% | 2561511054 |
| 16_Planctomycetes | 3.0 | 25 | 54.1 | 99.1% | 1.9% | 0.3% | 1.1% | 2588253524 |
| 17_Proteobacteria | 2.6 | 118 | 64.8 | 100.0% | 1.9% | 0.3% | 0.9% | 2622736602 |

^1)^: Estimated completeness and redundancy based on 105 EESCGs.

^2)^: relative abundance of genome bins in SE and LE metagenomes

1. Metabolic characteristics of isolated strains of *Anaerolineae*

| **Characteristic** | **Anaerolinea thermophila UNI-1^T^** | **Anaerolinea thermolimosa IMO-1^T^** | **Levilinea saccharolytica KIBI-1^T^** | **Leptolinea tardivitalis YMTK-2^T^** | **Bellilinea caldifistulae GOMI-1^T^** | **Longilinea arvoryzae KOME-1^T^** | **Thermanaerothrix daxensis**  **GNS-1^T^** | **Thermomarinilinea lacunofontalis**  **SW7^T^** | **Ornatilinea apprima**  **P3M-1^T^** | **Pelolinea**  **submarina**  **MO-CFXI^T^** |
| --- | --- | --- | --- | --- | --- | --- | --- | --- | --- | --- |
| **Morphology** | Filamentous | Filamentous | Filamentous | Filamentous | Filamentous | Filamentous | Filamentous | Filamentous | Filamentous | Filamentous |
| **Gram staining** | negative | negative | negative | negative | negative | negative | negative | negative | negative | negative |
| **Motility** | N | N | N | N | N | N | N | N | N | N |
| **Sporulation** | N | N | N | N | N | N | N | N | N | N |
| **Cell diameter, µm** | 0.2-0.3 | 0.3-0.4 | 0.4-0.5 | 0.15-0.2 | 0.2-0.4 | 0.4-0.6 | 0.2-0.3 | 0.2 | 0.3-0.7 | 0.13-0.15 |
| **Tmp range *,°C** | 50-60 (55) | 42-55(50) | 25-50 (37-40) | 25-50 (37) | 46-65 (55) | 30-40 (37) | 50-73 (65) | 37-65 (60) | 20-50 (42-45) | 10-37(25-30 7) |
| **PH range *** | 6.0-8.0 (7.0) | 6.0-7.5 (7.0) | 6.0-7.2 (7.0) | 6.0-7.2 (7.0) | 6.0-8.5 (7.0) | 5.0-7.5 (7.0) | 5.8-8.5 (7.0) | 5.5-7.3 (6.0) | 6.5-9.0 (7.5-8.0) | 5.5-8.5 (7.0) |
| **Doubling time**, h** | 72 (48) | 48 (10) | 56 (56) | 50 (50) | 45 (29) | 92 (38) | 100 (N/A) | 4.6 (N/A) | 6(6) | 37 (*) |
| **Major cellular fatty acids** | C16:0, C15:0, C14:0 | Ai-C17:0, i-C15:0, C16:0 | C14:0, i-C15:0, C16:0 | C17:0, C16:0, C14:0 | C16:0, C14:0, i-C15:0 | i-C15:0, ai-C15:0, C14:0 | C16:0, C18:0,  iso-C17:0, C20:0 | C12:0,C16:0, C16:1,C18:0, C18:1 | iso-C15:0,  anteiso-C 15:0 | C18:1ω 9, C16: ω 7,  C18 :0,C16:0 |
| **GC content,%** | 54.5 | 53.3 | 59.5 | 48.2 | 54.7 | 57.6 | 57.6 | 59.9 | 55 | 52.4 |
| **Yeast extract** | Y | Y | Y | Y | Y | Y | Y | **N** | Y | Y |
| **Arabinose** | ± | + | - | ± | + | - | ± | N/A | - | + |
| **Fructose** | + | + | + | + | + | ± | + | - | - | + |
| **Glucose** | + | + | + | + | + | - | + | - | + | + |
| **Sucrose** | + | + | + | + |  | + | + | - | + | + |
| **Raffinose** | + | + | + | + |  | + | + | N/A | N/A | + |
| **Pectin** | ± | ± | ± | + | + | + | N/A | - | - | + |
| **Tryptone** | ± | + | + | + | ± | + | N/A | + | N/A | - |
| **Xylose** | ± | + | + | + | ± | + | ± | - | + | + |
| **Xylan** | **±** | **±** | **±** | **+** | **±** | **+** | **+** | **-** | **-** | **+** |
| **Pyruvate** | ± | + | + | ± | + | - | + | - | **-** | - |
| **Starch** | + | ± | - | ± | - | - | - | - | **-** | + |
| **Cellulose** | N/A | N/A | N/A | N/A | N/A | N/A | N/A | N/A | **+** | N/A |
| **Acetate** | - | - | - | - | - | - | - | - | **+** | - |
| **Propionate** | - | - | - | - | - | - | - | - | N/A | - |
| **Butyrate** | - | - | - | - | - | - | - | - | N/A | - |
| **Growth products (minor products) ^1)^** | Ace,H_2_ (Lac, Suc, For) | Ace, Lac, H_2_ | Ace, For, H_2_ (Lac) | Ace, Lac, Pyr, H_2_ (Suc, For) | Ace, Lac, For, H_2_ (Pyr, Pro) | Ace, Lac, H_2_ | Ace, Lac | N/A | Ace, Eth, H_2_ (Lac,Pyr) | Ace, Lac, Eth, H_2_ (Pyr,Pro) |
| **Use of potential electron acceptor ^2)^** | -/-/-/- | -/-/-/- | -/-/-/- | -/-/-/- | -/-/-/- | -/-/-/- | -/-/-/- | -/-/-/- | -/-/-/- | -/-/-/- |
| **Isolation source** | Thermophilic anaerobic sludge | Thermophilic anaerobic sludge | Mesophilic anaerobic sludge | Mesophilic anaerobic sludge | Thermophilic anaerobic sludge | Rice paddy soil | Deep hot aquifer | Shallow sea hydrothermal vent | Deep terrestrial hot aquifer | Marine subsurface sediments |
| **Reference** | 1 | 2 | | | 3 | | 4 | 5 | 6 | 7 |

The characteristic features differed from rest of the strains were highlighted in yellow

*: Values in parentheses are the optimal growth temperature/pH

**: Values in parentheses are the doubling times recorded when the strains were co-cultured with hydrogenotrophic methanogens.

±: week growth;

N/A: information not available in the literature

1): sulphate/sulphite/nitrate/ Fe(III) as eclectron

2) :The abbreviations for the end products are as follows: Ace, acetate; Lac, lactate; Suc, succinate; Pyr, pyruvate; For, formate; Eth, ethanol; H 2 , hydrogen; Pro, propionate.

1. *Comparison of assembly by three different de novo assemblers. Only the SE metagenome were assembled for comparison.*

|  | MetaVelvet | IDBA_UD | CLCbio |
| --- | --- | --- | --- |
| Total Base (Mb) | 25.7 | 58.0 | 62.0 |
| Number of Scaffolds > 300 bp | 4,720 | 30,057 | 26,108 |
| N50 of Scaffolds > 300 bp | 28,993 | 10,951 | 8,643 |
| Percentage of reads in scaffolds >300 bp | 68.1% | **76.3%** | 79.0% |
| Number of Scaffolds > 1 kb | 2,852 | 7,444 | 10,611 |
| N50 of Scaffolds > 1 kb | 31,558 | **20,556** | 12,749 |
| Percentage of reads in scaffolds > 1 kb | 67.3% | 74.9% | 78.1% |
| Number of ORFs  from scaffolds > 300 bp | 28,600 | 78,633 | 83,279 |

1. Functional orthologues of genes putatively involve in electron transfer for syntrophic metabolism.

| Gene name | Accession | description |
| --- | --- | --- |
| **Pilus assembly protein *Flp/PilA (Pil*A)** | | |
| *pil*A | K02650 | type IV pilus assembly protein PilA |
| *flp*, *pil*A | K02651 | pilus assembly protein Flp/PilA |
| **c-type Outer membrane cytochrome (CytC)** | | |
| *Mtr*C | K00579 | tetrahydromethanopterin S-methyltransferase subunit C [EC:2.1.1.86] |
| *Mtr*C, *Omc*A | TIGR03507 | TIGR03507 (HMM): decaheme c-type cytochrome, OmcA/MtrC family |
| *Mtr*B/*Pio*B | TIGR03509 | TIGR03509 (HMM): decaheme-associated outer membrane protein, MtrB/PioB family |
| *Dms*E | TIGR03508 | TIGR03508 (HMM): decaheme c-type cytochrome, DmsE family |
| **Formate dehydrogenase (FDH)** | | |
| *fdo*G, *fdf*H | K00123 | formate dehydrogenase major subunit [EC:1.2.1.2] |
| FDH | K00122 | formate dehydrogenase [EC:1.2.1.2] |
| *fdh*A | K00148 | glutathione-independent formaldehyde dehydrogenase [EC:1.2.1.46] |
| *fdh*D | [K02379](http://www.kegg.jp/dbget-bin/www_bget?ko:K02379) | FdhD protein |
| *fdh*E | [K02380](http://www.kegg.jp/dbget-bin/www_bget?ko:K02380) | FdhE protein |
| *fdh*A1; | [K05299](http://www.kegg.jp/dbget-bin/www_bget?ko:K05299) | formate dehydrogenase alpha subunit [EC:1.2.1.43] |
| *fdh*B1 | [K15022](http://www.kegg.jp/dbget-bin/www_bget?ko:K15022) | formate dehydrogenase beta subunit [EC:1.2.1.43] |
| **Hydrogenase (Hyd)** | | |
| *hyd*B2 | [K00437](http://www.kegg.jp/dbget-bin/www_bget?ko:K00437) | [NiFe] hydrogenase large subunit [EC:1.12.2.1] |
| *hyd*A2 | [K18008](http://www.kegg.jp/dbget-bin/www_bget?ko:K18008) | [NiFe] hydrogenase small subunit [EC:1.12.2.1] |
| *hyd*A1 | [K17997](http://www.kegg.jp/dbget-bin/www_bget?ko:K17997) | iron-hydrogenase subunit alpha [EC:1.12.1.4] |
| *hyd*B1; | [K17998](http://www.kegg.jp/dbget-bin/www_bget?ko:K17998) | iron-hydrogenase subunit beta [EC:1.12.1.4] |
| *hyd*G1 | [K17999](http://www.kegg.jp/dbget-bin/www_bget?ko:K17999) | iron-hydrogenase subunit gamma [EC:1.12.1.4] |
| *hyd*B3 | [K05922](http://www.kegg.jp/dbget-bin/www_bget?ko:K05922) | quinone-reactive Ni/Fe-hydrogenase large subunit [EC:1.12.5.1] |
| *hyd*A3 | K05927 | quinone-reactive Ni/Fe-hydrogenase small subunit [EC:1.12.5.1] |
| *hyd*A; | [K17993](http://www.kegg.jp/dbget-bin/www_bget?ko:K17993) | sulfhydrogenase subunit alpha [EC:1.12.1.3 1.12.1.5] |
| *hyd*B | [K17996](http://www.kegg.jp/dbget-bin/www_bget?ko:K17996) | sulfhydrogenase subunit beta (sulfur reductase) [EC:1.12.98.4] |
| *hyd*D | [K17994](http://www.kegg.jp/dbget-bin/www_bget?ko:K17994) | sulfhydrogenase subunit delta [EC:1.12.1.3 1.12.1.5] |
| *hyd*G; | [K17995](http://www.kegg.jp/dbget-bin/www_bget?ko:K17995) | sulfhydrogenase subunit gamma (sulfur reductase) [EC:1.12.98.4] |
| *zra*R, *hyd*G | [K07713](http://www.kegg.jp/dbget-bin/www_bget?ko:K07713) | two-component system, NtrC family, response regulator HydG |
| *zra*S, *hyd*H | [K07709](http://www.kegg.jp/dbget-bin/www_bget?ko:K07709) | two-component system, NtrC family, sensor histidine kinase HydH [EC:2.7.13.3] |
| *Hyc*E | COG3260 | Ni,Fe-hydrogenase III small subunit |
| *Hyc*E | COG3261 | Ni,Fe-hydrogenase III large subunit |
| *Hyc*E | COG3262 | Ni,Fe-hydrogenase III component G [Energy production and conversion] |

1. Statistic of the post-QC HTS reads of 16S rRNA gene amplicons of the attachment samples

| Category | Sample name | Post QC reads number | Average read length | Number of OTUs | Shannon Index |
| --- | --- | --- | --- | --- | --- |
| Bacteria | Initial | 6534 | 436 | 203 | 4.8 |
|  | 1min | 14664 | 441 | 245 | 4.5 |
|  | 2h | 6377 | 438 | 175 | 4.4 |
|  | 6h | 6614 | 438 | 168 | 4.3 |
|  | 12h-1 | 7039 | 440 | 204 | 4.3 |
|  | 12h-2 | 14916 | 439 | 252 | 4.3 |
|  | 24h | 8488 | 445 | 270 | 4.1 |
| Archaea | Initial | 655 | 534 | 16 | 1.4 |
|  | 1min | 1257 | 528 | 27 | 1.9 |
|  | 2h | 457 | 486 | 23 | 2.4 |
|  | 6h | 929 | 502 | 26 | 2.2 |
|  | 12h-1 | 1128 | 484 | 36 | 2.4 |
|  | 12h-2 | 1640 | 518 | 36 | 2.2 |
|  | 24h | 884 | 508 | 41 | 2.3 |

1. Community structure of the TCF consortium showing the accumulation of Chloroflexi during long-term run of the enrichment SBR.

1. (a): Table showing the in-silico DNA-DNA hybridization values (DDH) (upper diagonal) and Average Nucleotide Identity (ANI) (lower diagonal) among five curated genomes retrieved and A. thermophila UNI-1 and C. aerophila DSM14535. Genomes in the table are ordered according to the phylogenetic relationship represented by the concatenated tree based on 35 shared ESCGs (to the left of the table). The number of aligned fragments used for ANI calculation is shown in bracket under the ANI value. (b) Venn diagram showing the number of shared and unique genes between TCF-8 and TCF-13 based on KEGG orthology annotation. (c) Venn diagram showing the number of shared and unique genes among TCF-2, 5, 12 and A. thermophila UNI-1 based on KEGG orthology annotation.

1. Hierarchy clustering of members of Chloroflexi based on Euclidean distance of COGs annotation of 32 available genomes of Chloroflexi phylum and five curated genomes retrieved. Finished genomes [F], permanent draft genomes [P] and draft genomes [D] were all considered to insure comprehensive functional comparison.

1. Transcriptional activities of genes involve in the Glycolysis pathway (partially shown) in the five curated bins and A. thermophila UNI-1. Filled blocks in the bottom and top role respectively represent genes encoded and expressed in the corresponding genomes Blocks are filled with the same position and color as the corresponding genomes in the legend.


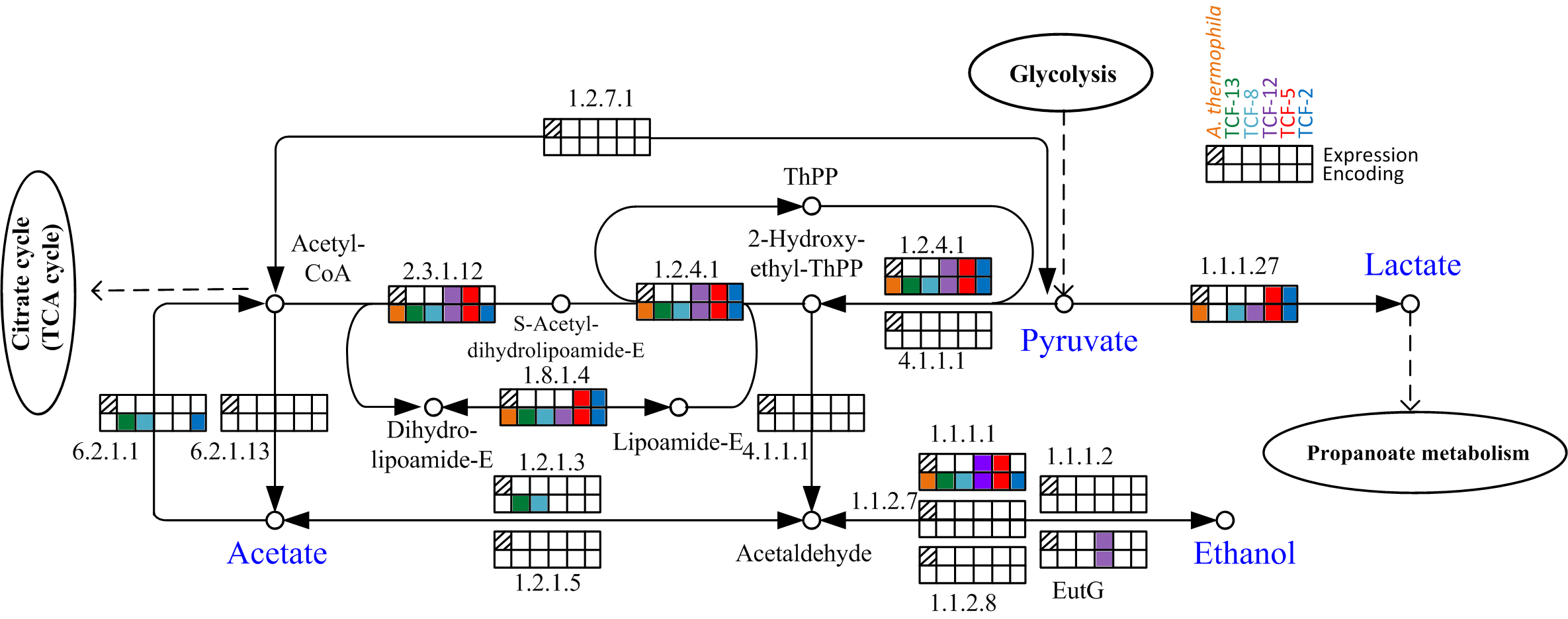


1. Rarefaction analysis of the community attached to cellulose surface. Top and bottom sub-tables respectively represent the rarefaction curve of Archaea (top) and Bacterial (bottom) community.

1. Comparison of methane (CH4) and major VFAs (acetic acid and propanoic acid) generation between Iron-supplemented (in form of Fe2O3) and control in consecutive batch tests. B1: the first batch; B3: the last consecutive batch.

1. Bacterial (left) and Archaea (right) community correlation between biological replicates sampled at twelve hours. Only prevalent populations taking >1% of the community are considered in correlation test.

1. Hydrolysis during attachment. The error bar represents the deviations between biological replicates sampled at twelve hours.

1. Electrophoretogram of the 16S rRNA genes amplicons used for high-throughput sequencing. Blank represents the band of filter paper. PCR products of 30 and 48 hours was not used in the sequencing. Takara DL2000 was used as marker.


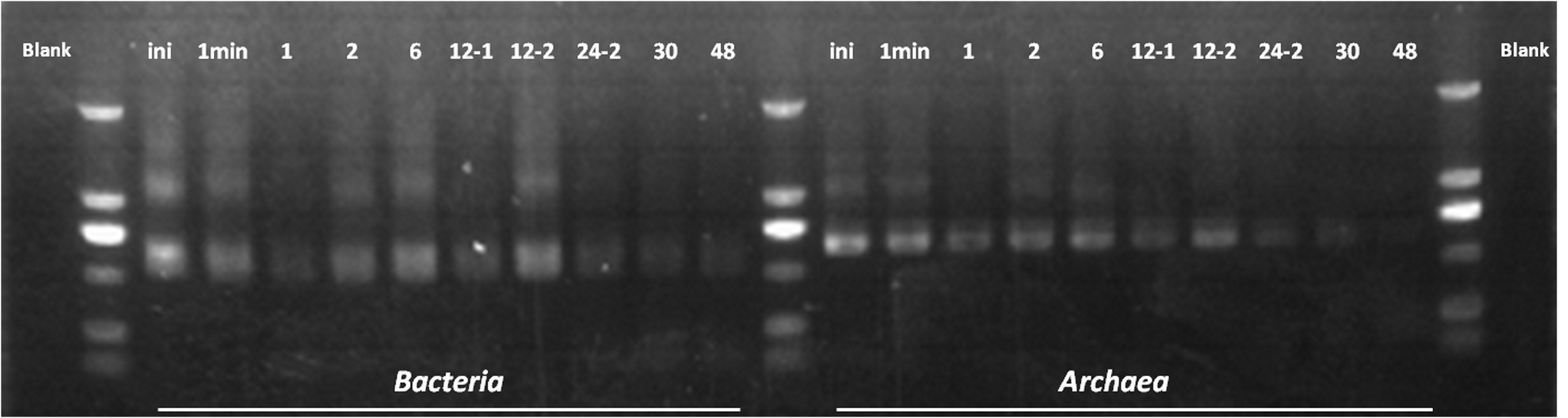


1. Composition of Archaea community during attachment. Only prevalent population taking > 1% in the community are shown in the figure.
